# Le mouvement mutualiste en Afrique entre nécessités, défis, espoirs et Covid-19

**DOI:** 10.48327/E6DW-5416

**Published:** 2021-02-18

**Authors:** J.-P. Boutin, J.-M. Milleliri

**Affiliations:** GISPE - Groupe d’Intervention en Santé Publique et Epidémiologie - Marseille, France

En Afrique plus de 60 % des dépenses de santé sont à la charge des ménages, lesquels trouvent leurs ressources à plus de 70 % dans le secteur informel. Dès lors la catastrophe sanitaire que constitue un accident de santé notable chez un adulte chargé de famille devient une catastrophe économique avant parfois de tourner à la catastrophe sociale.

La mutuelle de santé, construite sur un modèle importé du Nord, à but non lucratif, basée sur l’adhésion volontaire et la garantie d’accès à des paniers de services, constitue un moyen d’accès aux soins de plus en plus visible en Afrique. Comme tout système en pleine croissance, la mutualité africaine rencontre de nombreux défis qu’elle tente de franchir si possible simultanément plutôt que l’un après l’autre. La mise en place du Code de la mutualité de l’Union économique et monétaire ouest-africaine, et ses déclinaisons nationales, est une avancée majeure dans la nécessaire professionnalisation du mouvement mutualiste, mais dont les avancées pratiques sont encore loin de sauter aux yeux de l’adhérent de base qui compare son investissement personnel à la qualité des prestations reçues. Et quand survient une pandémie comme la Covid-19, l’édifice encore fragile, au modèle économique précarisé par le virus, au fonctionnement en rodage, en but à certains lobbys adverses, aux systèmes d’information débutant, les périls sont alors très grands. Heureusement la mutualité africaine n’est pas seule. Elle peut dorénavant s’appuyer sur un cadre multinational, la formalisation d’instances régulatrices nationales, des coopérations et partenariats avec les mouvements mutualistes européens et le soutien de l’Association internationale de la Mutualité (AIM). Ainsi en 2020, la crise de la Covid-19 qui aurait pu être douloureuse pour le mouvement mutualiste a permis à ce dernier de trouver sa place face au défi et de réaffirmer tout l’intérêt du « Pari de la mutualité ».

Le tout nouveau *Magazine* de la *Revue Médecine tropicale et santé internationale* (*MTSI*) inaugure aujourd’hui son premier feuilleton. Dès à présent, et tout au long des semaines à venir seront publiés une série d’articles, coordonnés par l’AIM, qui font le point sur les défis et les avancées récentes du mouvement mutualiste en Afrique de l’Ouest et Centrale. Chaque article aborde un aspect de l’actualité du sujet et l’ensemble forme un dossier cohérent. La rédaction du *Magazine* se félicite de l’aboutissement de ce projet éditorial en partenariat avec l’AIM. Le Groupe d’intervention en santé publique et épidémiologie (GISPE) qui anime la partie *Magazine* de la *Revue MTSI* souhaite ardemment que le mouvement mutualiste africain se développe et apporte à ses adhérents le soutien et le soulagement dont ils ont besoin au moment d’affronter les accidents de santé.

